# Compensation for Blur Requires Increase in Field of View and Viewing Time

**DOI:** 10.1371/journal.pone.0162711

**Published:** 2016-09-13

**Authors:** MiYoung Kwon, Rong Liu, Lillian Chien

**Affiliations:** Department of Ophthalmology, School of Medicine, University of Alabama at Birmingham, Birmingham, Alabama, United States of America; University of Leicester, UNITED KINGDOM

## Abstract

Spatial resolution is an important factor for human pattern recognition. In particular, low resolution (blur) is a defining characteristic of low vision. Here, we examined spatial (field of view) and temporal (stimulus duration) requirements for blurry object recognition. The spatial resolution of an image such as letter or face, was manipulated with a low-pass filter. In experiment 1, studying spatial requirement, observers viewed a fixed-size object through a window of varying sizes, which was repositioned until object identification (moving window paradigm). Field of view requirement, quantified as the number of “views” (window repositions) for correct recognition, was obtained for three blur levels, including no blur. In experiment 2, studying temporal requirement, we determined threshold viewing time, the stimulus duration yielding criterion recognition accuracy, at six blur levels, including no blur. For letter and face recognition, we found blur significantly increased the number of views, suggesting a larger field of view is required to recognize blurry objects. We also found blur significantly increased threshold viewing time, suggesting longer temporal integration is necessary to recognize blurry objects. The temporal integration reflects the tradeoff between stimulus intensity and time. While humans excel at recognizing blurry objects, our findings suggest compensating for blur requires increased field of view and viewing time. The need for larger spatial and longer temporal integration for recognizing blurry objects may further challenge object recognition in low vision. Thus, interactions between blur and field of view should be considered for developing low vision rehabilitation or assistive aids.

## Introduction

Spatial resolution is an important factor in human pattern recognition [1]. People often function near the spatial-resolution limit for pattern recognition. Imagine recognizing a familiar face across the street or recognizing letters on a distant traffic sign while driving. In these cases, spatial resolution is limited by visual acuity. It may be also limited by external factors, such as fog or low-resolution display rendering. On the other hand, low resolution (blur) is a defining characteristic of low vision, uncorrectable vision loss that interferes with daily activities, such as reading, recognizing objects, or driving [2]. Hence, dealing with blur is relevant to both normal and low vision.

Blur alters image information such that fine, local information (conveyed by low spatial frequencies) is reduced whereas coarse, global information (conveyed by high spatial frequencies) remains largely intact [3–6]. The question arises as to how human observers recognize an object when its featural information is severely degraded. Over the past several decades, a great deal of research has devoted attention to understanding the respective roles of the information carried by different spatial frequency bands (e.g., low vs. high) in object recognition [7–9] and the minimum or the most useful spectral information required for object recognition [6, 10–15]. A number of studies have shown that letter recognition relies on spatial frequencies between 1–3 cycles per letter [11, 13, 14, 16–20] whereas face recognition relies on spatial frequencies between 3–16 cycles per face [4–6, 12, 21–26]. Besides object type (e.g., letter vs. face), several other factors, including the object size [19, 27, 28] or the nature of task (e.g., identification vs. categorization) [5, 29, 30], also appear to influence spatial frequency requirements for object recognition. For instance, Majaj et al. [19] showed that the peak of the spatial-frequency band most useful for letter recognition shifted from 1.7 cycles per letter for small characters (0.16°) to 7.7 cycles per letter for large characters (16°). A similar size dependency was also found in face recognition [27, 28]. These findings suggest that for recognizing letters or faces, human observers would have higher tolerance for blur at smaller object sizes. Furthermore, enhancing luminance contrast appears to help people recognize blurry objects. Kwon and Legge [31] showed that the contrast recognition thresholds required for recognizing letters increased significantly with increasing blur (e.g., an increase by a factor of 8 from unfiltered letters to letters low-pass filtered with 0.9 cycles per letter cutoff spatial-frequency), suggesting the visual system increasingly relies on luminance contrast, as the spatial resolution of an object image is severely limited.

While these foregoing studies have provided us with a better understanding of human object recognition in the presence of blur, such studies measured observers’ recognition performance under conditions of unlimited viewing time and unrestricted field of view (full viewing), which might not reflect real-world experience. In the real world, the recognition speed of surroundings or objects is often critical (e.g., recognizing traffic sign while driving, searching for everyday objects). In addition, an observer’s field of view is often compromised due to either occlusion by other objects or sensory limitations in the observer’s visual field. In fact, it is not uncommon that visually impaired individuals exhibit deficits in both spatial resolution and field of view (e.g., AMD, diabetic retinopathy, retinitis pigmentosa, or glaucoma), highlighting the importance of studying these two properties together. Therefore, spatial and temporal requirements should be taken into account when we characterize human pattern recognition in the presence of blur.

A possible interaction between blur and field of view comes from an interesting observation: blur alters image information such that fine-grained featural information is diminished while configural information (e.g., the spatial interrelationship between local features revealed by global pattern of light) likely remains intact [3–6]. In other words, if blur eliminates high-resolution features, recognition must rely on the global attributes. For example, under normal viewing conditions, the fine featural details of the shape of eyes or nose can be useful for recognizing faces. However, this information might not be available under blurred viewing conditions. Observers would, thus, depend more on configural information that survives blur, such as the distance between eyes or the overall shape of face (e.g., elongated or round). Considering that a larger field of view is advantageous to access global or configural information, for a given size, recognizing blurry objects might need integration of visual information across a larger spatial extent (a larger field of view) compared to recognizing objects without blur. However, little is known about field of view requirement for recognizing blurry objects.

On the other hand, to recognize visual stimuli, the human visual system integrates visual information over a certain period of time. This temporal integration is often described by Bloch’s law, which contends the detectability of visual stimuli largely depends on their energy, the product of stimulus intensity (e.g., luminance) and duration [32]. While Bloch’s law holds mostly for light detection, evidence suggests that it could also be applied to detection of luminance contrast or complex shape recognition [33–36]. This reciprocal tradeoff between stimulus intensity and time would predict an increase in stimulus duration for object recognition as the spatial content of the object image becomes reduced (e.g., blur). This pattern of results has been suggested in Olds and Engel’s study [37] showing the difference in stimulus duration between low-pass filtered and unfiltered objects. While their findings are consistent with the foregoing prediction, more detailed work on the dependency of stimulus duration on the level of blur remains to be addressed.

Thus, the purpose of the current study was to examine spatial (field of view) and temporal requirements for object recognition in the presence of blur. Here, we asked the following two questions: (1) whether the field of view necessary for recognizing an object becomes larger as the spatial resolution of the object becomes reduced (i.e., larger spatial integration); (2) whether the threshold viewing time (i.e., stimulus exposure duration) that allows for reliable object recognition increases under conditions of blur (i.e., longer temporal integration).

In the first experiment, the field of view requirement for blurry object recognition was examined using a moving window paradigm. Observers viewed the target object through a viewing window (i.e., a circular aperture with varying size) which they were free to move over the target until they recognized its identity. This was achieved using a gaze-contingent display via a high-speed eye tracking system, so that the moving window always follows a participants’ gaze (i.e. gaze-contingent moving window) (we also verified our results using a mouse-tracking moving window). This paradigm measured visual field requirement for blurry object recognition by allowing us to assess the number of “views” (i.e., how many times observers had to reposition the viewing window across the target image) required to recognize the target object while varying the amount of information visible on the screen (i.e., window size). For given object and window sizes, when observers need to integrate visual features of an object over a greater spatial extent, we expected to see a greater number of views, an indication of larger field of view requirement. The measurements were made at three different blur levels, including no blur. The number of views at different blur levels was compared. A similar moving window paradigm had successfully been used in previous studies examining the minimum number of simultaneously visible characters (i.e., critical window size) necessary to achieve optimal reading performance [38–40]. Moving window paradigm has been also used with face, object, and scene stimuli to study mechanisms underlying perceptual and cognitive processes [41–44].

In a second experiment, the temporal requirement for blurry object recognition was examined by assessing threshold viewing time as a function of blur level. Observers’ recognition performance for face or letter stimuli under the full viewing condition (no restriction on field of view) was measured as a function of stimulus exposure time. Threshold viewing time was defined as the stimulus exposure time yielding criterion recognition accuracy (i.e., 80%), which represents the minimum stimulus exposure duration allowing for reliable letter and face recognition. Threshold viewing times obtained from six different blur levels were compared.

The outcome of these experiments is expected not only to help us to understand the perceptual process underlying object recognition in the presence of blur, but also to elucidate whether the spatial and temporal requirements for recognition of blurry objects play potential limiting factors on object recognition in low vision.

## Methods

### Participants

The current study comprised of four experiments: field of view study for letter or face; viewing time study for letter or face. A total of 30 participants took part in this study: eight participants for each field of view study (letter or face) and seven participants for each viewing time study (letter or face). All participants (mean age 24.70 ± 7.19 years; 11 males) were recruited from the University of Alabama at Birmingham campus between February 2014 and October 2015. They were all native English speakers with normal or corrected-to-normal vision and had no known cognitive or neurological impairments. The mean acuity (Lighthouse distance acuity chart) was -0.11 (± 0.11) logMAR and the mean log contrast sensitivity (Pelli-Robson chart) was 1.94 (± 0.07). Participants received monetary compensation. The experimental protocols were approved by the Internal Review Board (IRB) at the University of Alabama at Birmingham and written informed consent was obtained from all participants prior to the experiment.

### Stimuli and Apparatus

**Fig 1:**
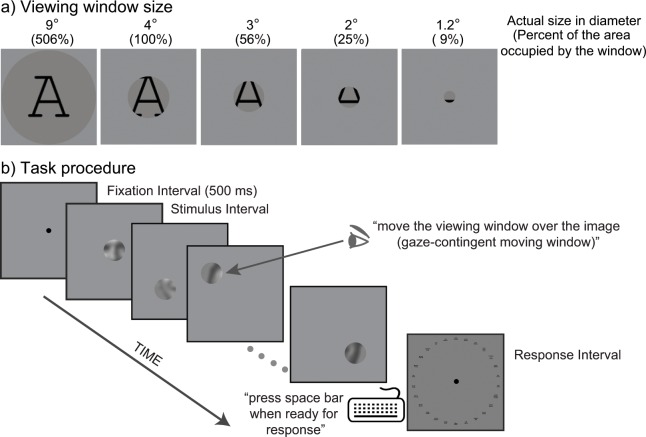
Schematic diagrams of stimulus and task procedure. **(a)** A target letter was viewed through an aperture with varying sizes (in diameter) ranging from 1.2° to 9° in diameter. The numbers in the parentheses indicate the aperture sizes as percentages of the area of the circle containing the stimulus target. The size of the target image was 4° of visual angle. **(b)** At the beginning of each trial, participants were instructed to fixate on a central dot on the display screen to make sure that the aperture always appears on the center of a target image. A participant’s task was to identify the target stimulus as quickly and accurately as possible. Participants freely moved the viewing window over the target image via a gaze-contingent display until they could recognize the target identity. Participants pressed space bar as soon as they recognized its identity. Then, participants reported the target identity by clicking one of 26-letter images or 26-face names response keys forming a clock face. This measurement was repeated for three different blur levels, including no blur.

Two common object types (letters and faces) were used to assess recognition performance (Fig 1). The spatial resolution of the object image was manipulated with a low-pass filter with different cutoff frequencies. Here, “low-resolution or blur” was defined as object spatial frequency (i.e., object-based resolution rather than absolute retinal acuity) and blur level was expressed as the cutoff spatial-frequency of the low pass-filter (cycles per object).

The 26 uppercase Courier font letters of the English alphabet were used for the letter recognition task. The letter images were constructed in Adobe Photoshop (version 8.0) and MATLAB (version 8.3). A single black letter was presented on a uniform gray background. Letter size, defined as x-height, was 4° (for the field of view study) or 2° (for the viewing time study) at a viewing distance of 57 cm.

Images of 26 well-known celebrities (13 females and 13 males) were used for the face recognition task. The faces were all smiling, viewed from the front, and without any conspicuous external cues, such as glasses, beards, or hair accessories. A single grayscale face was presented on a uniform gray background. The ranges of pixel gray-scale values of face images were similar across the 26 different face images: the minimum values ranged from 0 to 33 with median value of 1 and the maximum values ranged from 200 to 255 with median value of 241. The size of an image was determined by the edge-to-edge size of the face at eye level. The size of the face was 4° (for the field of view study) or 2° (for the viewing time study) at a viewing distance of 57 cm. All the 26 faces were scaled in size to equate them. The selection and description of the faces are provided in more detail by Kwon and Legge [13]. In the current study, all participants were shown the set of 26 faces prior to the experiment to confirm familiarity (only those who were able to recognize the faces with 100% accuracy were eligible to participate in face recognition tasks) and to inform the participants about the set of possible target faces.

The images were blurred using a 3rd order Butterworth low-pass filter in the spatial frequency domain. As summarized in Table 1, the cutoff spatial frequencies of the filter (in cycles per letter or face) ranged from 1.2 cycles per letter (c/letter) or 2.4 cycles per face (c/face) to 4 c/letter or 8 c/face. The blur levels and ranges for each object type (face or letter) and task (field of view or viewing time study) were chosen based on previous studies [13, 14] and our pilot data to make sure that the blur levels provide sufficient resolution to fit the psychometric function (i.e., an observer’ responses as a function of stimulus intensity).

**Table 1:** Cutoff spatial-frequencies of the low-pass filter used for the study.

**Field of view study**	**Letter (c/letter)**	1.5	-	-	-	2.8	Unfiltered
	**Face (c/face)**	5.2	-	-	-	8	Unfiltered
**Viewing time study**	**Letter (c/letter)**	1.2	1.4	1.8	2.2	4	Unfiltered
	**Face (c/face)**	2.4	3.2	4.4	6	8	Unfiltered

**Fig 2:**
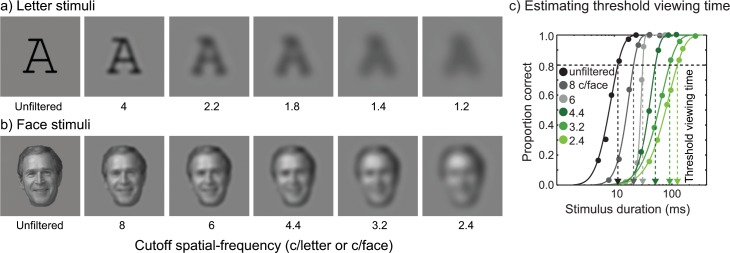
Sample stimulus images and illustration of psychometric functions. **(a)** Sample letter images with six different cutoff spatial frequencies ranging from 1.2 c/letter to 4 c/letter including unfiltered. **(b)** Sample face images with six different cutoff spatial frequencies ranging from 2.4 c/face to 8 c/face including unfiltered. The size of target letter or face was 2° of visual angle. **(c)** Estimating threshold viewing time from a psychometric function. For a given cutoff spatial-frequency, object recognition performance was measured as a function of stimulus duration (msec). Six different exposure durations were interleaved within a block. Each duration was repeated 30 times, resulting in a total of 180 trials for each psychometric function. Target object recognition was tested for each duration and percent correct recognition was computed at each stimulus duration. The resulting data (solid circles) were fit with Weibull functions [51] to derive a threshold viewing time (dotted arrow line) defined as the stimulus duration yielding 80% recognition accuracy (black dashed line). The solid line is the best fit of the function. A psychometric function was obtained for each cutoff spatial-frequency, amounting to six psychometric functions for each participant.

The filter function is

$$f=\frac{1}{\left(1+{\left(\frac{r}{c}\right)}^{2n}\right)},$$ (1)

where *r* is the radial frequency, *c* is the cutoff spatial frequency and *n* is the filter’s order. Fig 2 shows sample letters (a) and faces (b) with different blur levels.

To present the filtered images on the monitor, we mapped the luminance values of the letters to the 256 gray levels. The DC value of the filtered image was always mapped to the gray level of 127, equivalent to the mean luminance of the monitor (67 *cd/m*^*2*^*)*. The stimuli were generated and controlled using MATLAB (version 8.3) and Psychophysics Toolbox extensions [45, 46] for Windows 7, running on a PC desktop computer (model: Dell Precision Tower 5810). Stimuli were presented on a liquid crystal display monitor (model: Asus VG278H-E; refresh rate: 144 Hz; resolution: 1920×1080, subtending 60°×34° visual angle at a viewing distance of 57 cm). Luminance of the display monitor was made linear using an 8-bit look-up table in conjunction with photometric readings from a MINOLTA LS-110 Luminance Meter (Konica Minolta Inc., Japan).

### Eye Movement Recording and Moving Window

Participants’ eye movements were monitored (monocular tracking) using an infrared video-based eye-tracker sampled at 500 Hz (EyeLink 1000 Plus/Desktop Mount, SR Research Ltd., Ontario, Canada) with a maximum spatial resolution of 0.01°. A 9-point calibration/validation sequence was performed at the beginning of every experimental session that relied on the eye-tracker. Calibration and/or validation were repeated until the validation error was smaller than 0.5° on average. The gaze position error, the difference between the target position and the computed gaze position, was estimated during the 9-points validation process. The average gaze position error was 0.2°. A real-time gaze position was sent to the display computer through a high speed Ethernet link. The continuous gaze information was used to draw a viewing window on the display screen at a refresh rate of 144Hz.

Gaze data were analyzed using the EyeLink parsing algorithm, which robustly classified fixations and saccades, excluding blinks. The saccadic velocity threshold of 30°/sec, saccadic acceleration threshold of 8000°/sec^2^, and saccadic motion threshold of 0.1° were used to define saccades from fixations [47–50].

### Procedure

For both field of view and viewing time studies, letter recognition and face recognition were conducted in separate experiments on different participant groups. Both experiments, however, used the same procedure. All the testing was done in a dimly lit room. A chin-rest was used to reduce head movements and maintain viewing distance.

#### Field of view requirement for blurry object recognition

In each trial, participants were presented with a stimulus letter (A~Z) or face (one of 26 celebrity faces) masked by a layer with the same luminance and color as the background. A circular aperture (a “viewing window”) was always present in the layer and its location was controllable by eye movements (Fig 1). For a given trial, the size of the target object was fixed at 4° visual angle, while the size of a viewing window (in diameter) was selected randomly from given sizes: 1.2°, 2°, 3°, 4°, and 9° visual angle. The target stimulus was presented in the middle of the display screen. At the beginning of each trial, participants were asked to fixate on a central dot on the display screen to make sure the aperture always appears on the center of a target image. This was done to minimize any positional bias. Participants viewed the target through the window. Their task was to identify the target stimulus as quickly and accurately as possible. Participants were instructed to move their gaze freely over the target image, so that they could examine different features (parts) of the target image (Fig 1B). Once observers recognized the identity, they were told to press space bar as quickly as possible, which set the display to average luminance. After a brief pause (500 msec), a set of 26 thumbnail versions (56×56 pixels in size) of letter images (for the letter recognition) or a list of 26-target-face’s names (for the face recognition) appeared on the screen in a clock face. Then, participants reported the target identity by clicking one of 26-letter images or 26-face names response keys (Fig 1B). To prevent participants from using any image matching strategy, a different font (Arial) for letter or celebrities’ names for face were used for the response key. The letter images shown in the response key were unfiltered ones. The location of each response item on the response key was shuffled every block to avoid any response bias induced by specific location.

For each window size, we recorded the number of times observers moved the viewing window to different locations. These measurements were made for three different blur levels: 1.5 c/letter, 2.8 c/letter, and no blur for letter; 5.2 c/face, 8 c/face, and no blur for face (Table 1). The three blur levels were measured in separate blocks, and the set of blur levels were repeated twice: one in ascending and the other in descending order. Thus, 6 blocks were performed in each task (letter or face recognition) and each block consisted of 80 trials (16 trials × 5 window sizes). Participants were given a series of practice trials before the experimental test.

As participants performed the task, response times and window movements were recorded. In this study, we quantified the field of view as the total number of “views” required for correct recognition (only correct trials were analyzed in this study). The number of “views” was obtained from participants’ movement of the window via the gaze-tracking system. Gaze-tracking data (sampling rate of ~500Hz) were processed by the Eyelink parsing algorithm to robustly classify a “view” (i.e., act of acquiring visual information by repositioning the viewing window across the target image), analogous to a fixational eye movement, and a “pass-by”, analogous to saccadic eye movement. A detailed explanation of eye movement recording and data analysis was described in the Method section. We also verified our results by using a mouse tracking system in which participants moved the viewing window via a mouse, which is believed to be immune to idiosyncratic characteristics of eye movements. Despite the obvious methodological differences, the pattern of results qualitatively agreed with each other (see Discussion for more details).

#### Temporal requirement for blurry object recognition

The method of constant stimuli was used to present target objects at six stimulus durations in logarithmically spaced steps, spanning ~0.85 log units. The testing session was preceded by a practice session. During this session, the range of stimulus durations for each participant was chosen to make sure that at least 90% correct response was obtained at the longest stimulus duration and at most 20% correct response was obtained at the shortest stimulus duration. Participants initiated each trial by pressing a key. The target objects were presented for a given duration, followed immediately by a phase-scrambled mask (3° width × 3° height) which lasted for 500 msec. Then, a set of 26 thumbnail versions (56 × 56 pixels in size) of letter images (for the letter recognition) or a list of 26-target-face’s names (for the face recognition) appeared on the screen in a clock face. Participants reported the target object identity by clicking one of 26-letter images or 26-face names response keys. A different font (Arial) or celebrities’ names were used for the response key in order to prevent participants from using any image matching strategy.

Target object recognition was tested for each stimulus duration and percent correct recognition was computed at each stimulus duration. Psychometric functions, plots of percent correct recognition as a function of stimulus duration, were created by fitting these data with Weibull functions [51] as shown in Fig 2C. Threshold viewing time was defined as the stimulus duration yielding 80% correct responses (Fig 2C). Trials with six different durations were randomly interleaved within a block. Each duration was presented 30 times, so there were 180 trials for each psychometric function.

These measurements were made for six different blur levels, including no blur: 1.2 c/letter, 1.4 c/letter, 1.8 c/letter, 2.2 c/letter, 4 c/letter, and no blur for letter; 2.4 c/face, 3.2 c/face, 4.4 c/face, 6 c/face, 8 c/face, and no blur for face (Table 1). Threshold viewing time for each blur level was measured in a block. The order of blocks was counterbalanced across participants. Participants were given a series of practice trials before the experimental test.

## Results

### An increase in required field of view with increasing blur level

**Fig 3:**
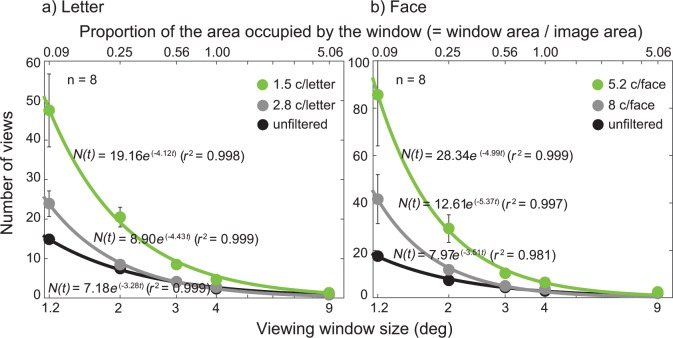
The results from the viewing window study for letter and face. The number of views was plotted as a function of window size (°) for three different blur levels: unfiltered (black dots), 2.8 c/letter or 8 c/face (gray dots), and 1.5 c/letter and 5.2 c/face (green dots). Stimuli were 26 uppercase letter **(a)** or 26 celebrity faces **(b)** with the image size of 4° of visual angle. The percent of the area occupied by the moving window with respect to the area of the target image was shown on the upper x-axis. Each data point (solid dots) was an average of the number of views across participants (*n* = 8). Data were fitted with the exponential-decay function (Eq 2). The solid lines are the best fits of the model. Error bars represent ±1 Standard Errors of the Mean (SEM).

Fig 3 plots the number of views as a function of window size for three different blur levels. The figure also shows normalized window size on the upper x-axis, calculated from window area divided by the target image area. Five different window sizes (1.2°, 2°, 3°, 4°, and 9° in diameter) were used for letter (Fig 3A) and face (Fig 3B). Each data point was an average of the number of views across eight participants. The data were fitted with Eq 2, the following exponential decay function:

$$N(t)=N_{0}e^{-\lambda t},$$ (2)

where *N*_0_ is the initial value, λ is the decay rate and *t* is log window size in degree. Overall, the model fits were satisfactory, with *r*^*2*^ values of 0.98 to 0.99, indicating that approximately 98~99% of variance can be accounted for by the exponential decay model.

As expected, the number of views increased as the window size decreased for letters with no blur (black solid dots in Fig 3). This pattern was well characterized by the exponential decay function (Eq 2). However, the increase in the number of views was more pronounced with increasing blur level, manifested in a larger parameter value of *N*_0_ and/or λ for the model fit. For example, the average number of views for letters with no blur increased from 1 at 9° window size to 15 at 1.2° window size, while the number for 1.5 c/letter blur level increased from 1 at 9° window size to 48 at 1.2° window size. A similar pattern was observed in faces, except that the differences in the number of views between no blur and blurred conditions were even greater. Our results showed that when the window size was large enough to reveal the entire object, the number of views required to identify the target object was not significantly different among three blur levels. However, when the window size became increasingly small, the difference in the number of views among three blur levels grew larger, indicating that recognizing blurry object requires larger spatial integration.

It is also noteworthy that for objects with either no blur or the blur level of 2.8 c/letter or 8 c/face, object recognition remained independent of the window size until the window size was reduced down to 3° (i.e., 56% of the target area). In contrast, for letters with the blur level of 1.5 c/letter or faces with the blur level of 5.2 c/face, the number of views became already significantly greater even with the window size of 3° (i.e. 56% of the target area). These results suggest that the critical window size (i.e., the window size that allows for object recognition with a single view) increases as the spatial resolution of an object image becomes significantly limited.

We performed an analysis of variance (ANOVA) on the number of views required to recognize letter reliably (i.e., 80% accuracy)– 3 (blur level: 1.5 c/letter, 2.8 c/letter, and no blur)×5 (window size: 1.2°, 2°, 3°, 4°, and 9°) repeated measures ANOVA with blur level and window size as within-participant factors. As expected, there was a significant main effect of window size (*F*_(4, 98)_ = 52.20, *p* < 0.001), indicating that more views are required to recognize letters as the window size decreases. More importantly, we found a significant main effect of blur level (*F*_(2, 98)_ = 21.66, *p* < 0.001) on the number of views, indicating that a larger field of view is needed to recognize blurry letters compared to less blurry ones. We also found a significant interaction effect between blur level and window size (*F*_(8, 98)_ = 6.54, *p* < 0.001), suggesting that the difference in the number of views between blurred and less burred letters is greater for smaller window sizes. We also performed a 3 (blur level: 5.2 c/face, 8 c/ face, and no blur) × 5 (window size: 1.2°, 2°, 3°, 4°, and 9°) ANOVA on the number of views required to recognize face. We found the same pattern of results as letter stimuli. There was a significant main effect of window size (*F*_(4, 98)_ = 26.63, *p* < 0.001). We found a significant main effect of blur level (*F*_(2, 98)_ = 12.63, *p* < 0.001) on the number of views, demonstrating that there should be an increase in field of view to recognize blurry faces compared to less blurred ones. We also found a significant interaction effect between blur level and window size (*F*_(8, 98)_ = 4.97, *p* < 0.001), suggesting that the difference in the number of views between blurred and less burred faces is greater for smaller window sizes.

**Fig 4:**
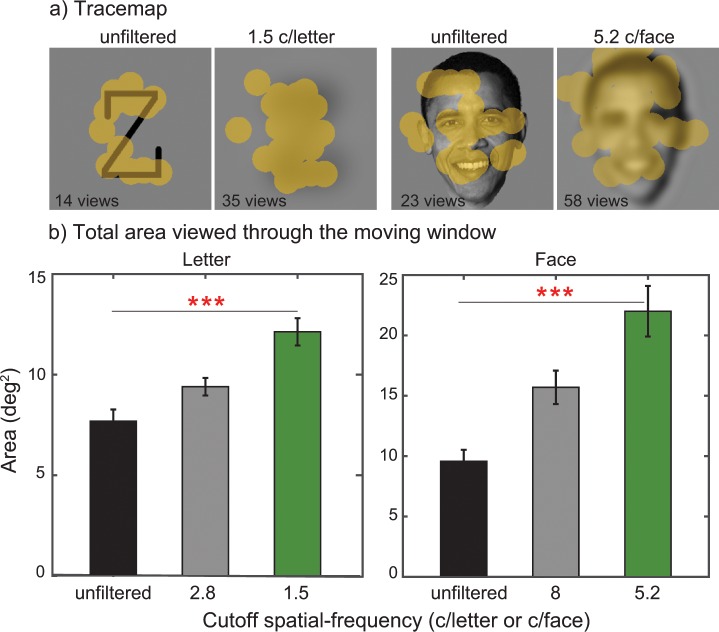
Examples of traces of the gaze-contingent moving window and the total area viewed by the moving window. **(a)** These trace maps show example trials (correct trials) taken from letter or face recognition for unfiltered and the lowest cutoff spatial-frequency (1.5 c/letter for letter and 5.2 c/face for face). The traces of the moving window (yellow blobs) were superimposed on target images. **(b)** Mean total amount of viewed area (deg^2^) collapsed across participants (*n* = 8) was plotted as a function of cutoff spatial-frequency (blur level) for letter and face. Area data were computed by aggregating the regions (e.g., yellow blobs in Fig 4A) of an object image viewed through the moving window (1.2°). Error bars represent ±1 SEM. Note that three asterisks (***) indicate the p value of < 0.001.

Fig 4A shows examples of traces of the gaze-contingent moving window for unfiltered objects and objects with the lowest cutoff spatial-frequency (1.5 c/letter for letter recognition or 5.2 c/face for face recognition) using the smallest window size (1.2°). The yellow blobs indicate the positions of the moving window superimposed on the target image. The number of views is also denoted in each figure. As demonstrated in these examples, the positions of the moving window seem to be clustered around features of an object (e.g., junctions between two lines for letter recognition or eyes or mouth area for face recognition) under no blur viewing while its positions appear to be spread over the entire image under blur viewing, requiring integration of visual information over a larger spatial extent. Consistent with our prediction, this pattern indicates access to configural information is likely to be more necessary for blurry object recognition compared to unblurred object recognition of the same size. It should be also noted that the importance of features such as the eyes or mouth in the processing of human faces has been reported in previous studies of face recognition [52–57].

To see if the total area of an object image viewed through the moving window was indeed larger for blurrier objects than for less blurry objects, we further quantified the field of view requirement by summing up the regions of an object image viewed through the gaze-contingent moving window (e.g., the area of yellow blobs in Fig 4A) across images. Fig 4B shows a plot of the estimated total area as a function of blur level for letter and face. We found that there was a significant increase in viewed area with increasing blur for both letter (*F*_(2,14)_ = 32.153, *p* < 0.001) and face recognition (*F*_(2,14)_ = 30.367, *p* < 0.001), suggesting that our indirect measure using the number of views reflects the field of view requirement for blurry objects.

### An increase in threshold viewing time with increasing blur

**Fig 5:**
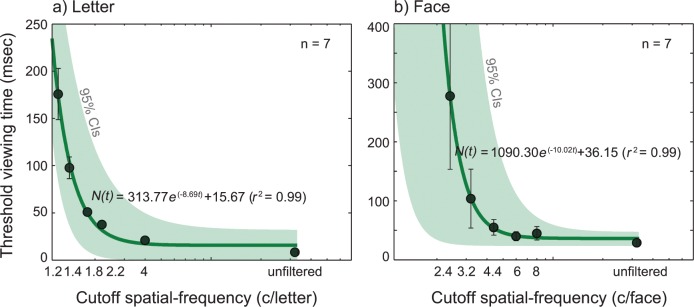
The results from the viewing time study for letter and face. Threshold viewing time (msec) was plotted as a function of cutoff spatial-frequency (blur level). Threshold viewing time was defined as a stimulus exposure time that yielded 80% recognition performance. Stimuli were 26 uppercase letter **(a)** or 26 celebrity faces **(b)** with the image size of 2° of visual angle. Each data point (black solid dots) was an average of threshold viewing time across participants (*n* = 7). Data were fitted with the exponential-decay function (Eq 3). The solid lines are the best fits of the model. Error bars represent ±1 SEM. The green shaded areas indicate 95% Confidence Intervals of the fit.

Fig 5 plots the mean threshold viewing time (msec) as a function of blur level for letter **(a)** and face **(b)**. Table 2 summarizes mean and standard errors for threshold viewing time for six blur levels for letter and face.

**Table 2:** Mean threshold viewing time as a function of cutoff spatial-frequency for letter and face.

**Cutoff spatial-frequency for Letter (c/letter)**		**1.2**	**1.4**	**1.8**	**2.2**	**4**	**Unfiltered**
**Threshold viewing time (msec)**	Mean	175.81	97.76	51.00	37.66	21.02	8.40
	SEM	±27.10	±11.51	±2.79	±2.50	±1.46	±0.62
	Mean	277.48	103.77	55.04	39.86	44.70	29.01
	SEM	±124.24	±49.83	±13.14	±7.59	±11.45	±6.33
**Cutoff spatial-frequency for Face (c/face)**		**2.4**	**3.2**	**4.4**	**6**	**8**	**Unfiltered**

*Note* that SEM refers to Standard Errors of the Mean.

As shown in Fig 5, the mean threshold viewing time for letters significantly increased from 8.40 msec (± 0.62) at no blur to 175.81 msec (± 27.10) at the 1.2 c/letter blur level. For faces, it increased from 29.01 msec (± 6.33) at unfiltered to 277.48 msec (± 124.24) at 2.4 c/face blur level (all *p*s < 0.05). The threshold viewing time for object recognition remained independent of stimulus blur level up to the cutoff spatial frequency of approximately 2 c/letter for letter or 4 c/face for face, while the viewing time became increasingly larger as stimulus became severely blurred.

For both object types, the relation between threshold viewing time and blur level was well characterized by Eq 3, the following exponential decay function:

$$N(t)=N_{0}e^{-\lambda t}+c,$$ (3)

where *N*_*0*_ is the initial value, λ is the decay rate, *c* is constant and *t* is log blur level in cycles per object. Overall, the model fits were satisfactory with *r*^*2*^ value of 0.99 for both letter and face data, indicating that 99% of variance is accounted for by the exponential-decay model.

One-way repeated measures ANOVA compared the means of threshold stimulus duration for recognizing letters or faces at six blur level conditions. We found a significant main effect of blur level on threshold viewing time (msec) for recognizing letters (*F*_(5, 30)_ = 30.01, *p* < 0.001) or faces (*F*_(5, 30)_ = 3.98, *p* = 0.007), indicating a longer viewing time is necessary to recognize objects as the spatial contents of the object image becomes severely reduced. These results suggested that the human visual system does recognize severely blurry objects (e.g., 1.2 c/letter or 2.4 c/face) with high accuracy (80%), yet a longer viewing time is required to perform reliable recognition performance with increasing blur. It is also noteworthy that the blur effect reached an asymptote around the cutoff frequency of 2 c/letter or 4 c/face, suggesting that object recognition becomes independent of viewing time as the blur level of objects exceeds these cutoff frequencies.

**Fig 6:**
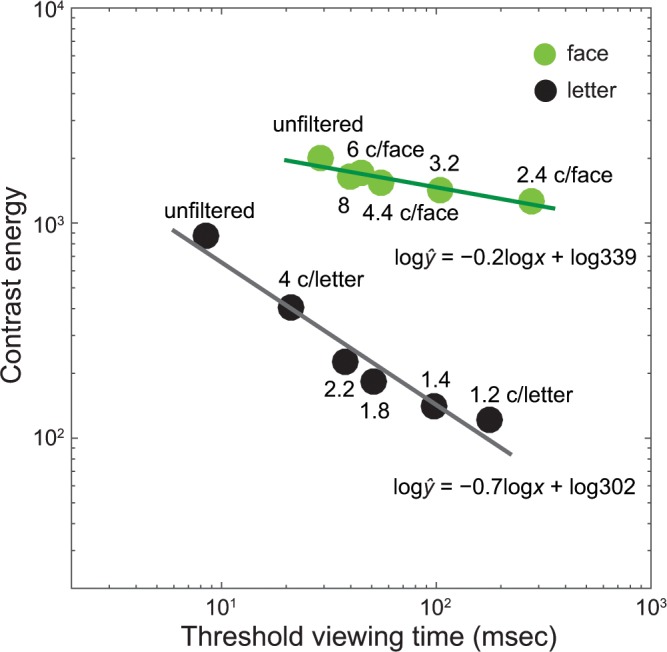
Contrast energy as a function of threshold viewing time (msec) for face and letter. Contrast energy of each image was computed as follows: $\sum_{i=1}^{N}\sum_{j=1}^{M}{\left((L_{i,j}-\bar{L})/\bar{L}\right)}^{2}$, where *L*_*i*, *j*_ is the luminance of the pixel [*i*, *j*] of an image of size *N* by *M*, and $\bar{L}$ is the mean luminance of the stimulus image. Each data point (solid dots) was an average of threshold viewing time across participants (*n* = 7) for a given blur level. The solid lines are the best fits of a linear function to the data.

We then asked whether this pattern could be described by Bloch’s law, specified as the reciprocity of stimulus intensity and duration. To this end, we computed stimulus contrast energy for each blur level and constructed a plot of contrast energy versus threshold viewing time (msec) for both letter and face (Fig 6). The intensity-time reciprocity would predict a negative slope (−1) of a simple linear function in logarithmic coordinates. As demonstrated in Fig 6, there was a trade-off between intensity and time for both letter (a slope of −0.7) and face (a slope of −0.2) recognition, suggesting that the longer temporal integration for blurry object recognition may be in part explained by the front-end temporal filtering properties of the human visual system.

## Discussion

Here, we studied 1) whether/how visual field requirement for blurry object recognition is different from no-blur viewing condition; 2) whether/how the threshold viewing time that allows for reliable object recognition (80% accuracy) depends on the spatial resolution of objects.

By limiting the information simultaneously visible using a viewing window paradigm, we measured the minimum number of “views” required for observers to recognize object (letter or face) at different blur levels. We expected, regardless of blur level, the number of views would increase as we decrease the viewing window size, which was exactly what we found. When the window size (9°) was larger than the target image (4°), observers made approximately one view before they identified the target, meaning that they did not have to move their viewing window. This pattern held true for both blur and no blur conditions. On the other hand, when the window size became considerably smaller than the target (<25% of the target size), observers moved the viewing window over the target image many times until they identified the target. This pattern helped us validate our gaze tracking data analysis.

**Fig 7:**
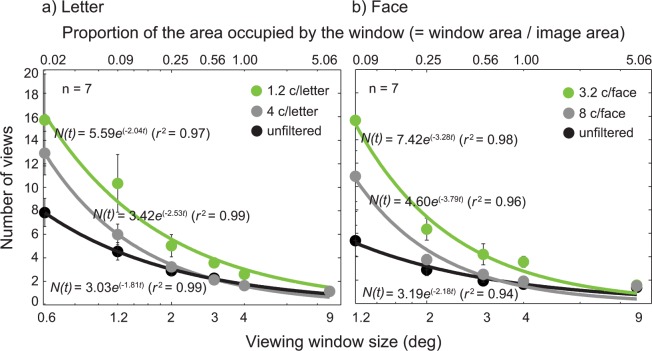
The results from the mouse tracking moving window. The number of views was plotted as a function of window size (°) for three different blur levels: unfiltered (black dots), 1.2 c/letter or 3.2 c/face (gray dots), and 4 c/letter and 8 c/face (green dots). Stimuli were 26 uppercase letter **(a)** or 26 celebrity faces **(b)** with the image size of 4° of visual angle (the same format as in Fig 3). Each data point (solid dots) was an average of the number of views across participants (*n* = 7). Data were fitted with the exponential-decay function (Eq 2). The solid lines are the best fits of the model. Error bars represent ±1 SEM.

We, however, acknowledge that our method of using the gaze-contingent moving window is an indirect way of measuring the field of view requirement for object recognition. For this reason, we first checked to see if the area of an object image viewed through the moving window was indeed larger for blurry objects than less blurrier objects. We tested this idea by summing up the regions of an object image viewed through the gaze-contingent moving window (1.2° window size). The total viewed areas of the three blur levels were compared. As demonstrated in Fig 4B, we found that there was a statistically significant increase in the total amount of viewed area with increasing blur, suggesting that our indirect measure via the number of views reflects the field of view requirement for recognizing blurry objects (remember that only correct trials were analyzed). However, it is still possible that the observed pattern of results was largely due to idiosyncratic characteristics of eye movements. To address this issue, we further verified our results by using a mouse tracking moving window in which participants moved the viewing window via a mouse. Stimuli and procedure were identical to the gaze-contingent moving window experiment. Fig 7 summarizes the results obtained with the mouse tracking moving window. Despite the methodological differences, we found that the same pattern (i.e., the larger number of views are needed for recognizing blurry objects compared to unblurred objects) as the gaze-contingent moving window. For example, the number of views for letters with no blur increased from 1 at 9° window width to 8 at 0.6° window width, while the number for 1.2 c/letter blur level increased from 1 at 9° width to 16 at 0.6° width. The findings obtained from the mouse tracking moving window together with the results from the total viewed area analysis convinced us that although the absolute number of views may change depending on experimental methods, the key pattern of results (larger field of view requirement for blurry object recognition) likely remains unchanged.

Now, going back to our main question, we looked at whether the number of views would increase with increasing blur level, an indicator of the larger visual field requirement (larger spatial integration) for blurry object recognition. We found, as blur level increased, the number of views to recognize an object increased more rapidly with decreasing window size, suggesting that recognizing blurry objects indeed requires a larger field of view. What could account for this phenomenon? Perhaps under blur viewing conditions, the visual system might have to rely on different visual features to identify objects. It is known that blur diminishes finer grained information about the object while maintaining global or configural information (i.e., global pattern of light). Because global or configural information is likely spread over a larger spatial extent of the visual field compared to fine details of visual features, the system might have to integrate visual information across a larger spatial extent in order to access the global information under blurry viewing conditions.

We next asked whether/how the threshold viewing time (i.e., stimulus duration) increases for blurry objects (longer temporal integration). By measuring recognition performance as a function of stimulus duration, we obtained the threshold viewing time corresponding to criterion recognition accuracy (80%) for each blur level. Our results showed that the human visual system does recognize considerably blurry objects (e.g., 1.2 c/letter or 2.4 c/face) with high accuracy, yet longer temporal integration is required to perform reliable object recognition as its blur level increases. The plot of threshold viewing time as a function of blur level was well characterized by the exponential-decay function (see Eq 3). For example, while stimulus durations of 8.40 msec and 29.01 msec appeared to be sufficient for observers to recognize familiar letter or face in high resolution, the exposure duration had to be increased by a factor of 21 for the most blurry letter (1.2 c/letter) or by a factor of 10 for the most blurry face (2.4 c/face) to achieve the same recognition performance.

Previous studies, using stimuli with normal spatial resolution, have also demonstrated that human observers are capable of getting the ‘gist’ of complex images even when the stimulus is presented as briefly as 10 or 20 msec (stimulus exposure duration) (for a review see Hegde [58]). However, it usually takes 150 msec up to 200 msec for them to recognize objects or scenes (recognition time) [58–63]. While there is vast literature on temporal dynamics of object recognition, little attention has been paid to temporal processing of blurry object recognition. To our knowledge, Olds and Engel [37] is the only study that reported differences in stimulus duration between low-pass filtered and unfiltered objects. Consistent with its findings, our results further characterized the temporal dependency of blurry object recognition across a wider range of blur levels. Our results also demonstrated that the critical stimulus duration required for reliable object recognition differs between letter and face. For no blur condition, observers required much longer stimulus exposure durations for face compared to letter (8.40 msec and 29.01 msec on average, *p* < 0.01). This pattern still held for blurred conditions, even when overall blur level used for face images was much less severe. Perhaps this is due to the dependence of the minimum exposure duration on object complexity. More complex object categories, such as faces, demand a longer exposure duration than simple and overlearned objects, such as letters.

Why do blurry objects require more time to recognize? As shown in Fig 6, we observed the reciprocal tradeoff between stimulus intensity and time. Although speculative, it thus appears that either this type of temporal integration or the limitations in the front-end visual processing, such as spatial-temporal filtering properties of human vision, might have influenced our results. Movshon [64] examined the effect of temporal modulation on low spatial frequency contrast sensitivity tuning functions in a cat’s striate cortex using single-cell recording techniques. They found that the overall spatial contrast sensitivity function decreased considerably with increasing temporal frequency, manifested by a downward shift of the sensitivity function. For example, while there was no significant difference in overall contrast sensitivity between the temporal frequencies of 1 Hz and 4 Hz, there was a considerable reduction (by a factor of 6) in overall contrast sensitivity (i.e., the peak value of the tuning function) when the temporal frequency of 16 Hz was applied. This suggests longer temporal requirement for low spatial-frequency inputs. The studies of human spatial-temporal contrast sensitivity functions done by Kelly [65, 66] provide further evidence that, compared to intermediate spatial frequencies, there was a much steeper decline in contrast sensitivity for lower spatial frequencies when temporal frequency became increasingly high.

While this reduced sensitivity for low spatial frequency inputs is likely to delay the initial image-driven, feed-forward sweep stage of visual processing, the possible account of later stages of visual processing should be also considered. For example, our field of view study demonstrated that to be able to recognize blurry objects, it is necessary to integrate visual information over a larger spatial extent. From an information theory perspective [67], a critical amount of information (in bits of information) needs to be transmitted along the visual pathways for the system to achieve criterion “recognizability” or “discriminability.” If an object image becomes increasingly blurry, the amount of information contained in a given local region is likely to be diminished (due to an increase in noise and/or a reduction in the relevant signal). In this case, the system might have to compensate its loss by pooling input signals from larger areas of visual space (i.e., an increase in spatial summation area). It is, thus, possible that the need to integrate visual information over a larger visual field might in part account for the longer temporal requirement for blurry objects. Furthermore, relying more on top-down feedback for discriminating visually degraded inputs (blurry objects) and such recurrent processing between early visual cortical areas and higher ventral stream areas might have demanded longer temporal requirement for recognizing blurry objects. Despite these speculations, the exact mechanism underlying the longer temporal integration for blurry objects remains to be answered.

We acknowledge that possible interactions between task demand and stimulus information should be taken into account before generalizing our findings to other behavioral contexts. Observers of the current study were asked to identify one letter (or face) out of 26 alternatives. For instance, fine featural information such as mouth or eyes might be critical for identifying 1 out of 26 alternative faces. However, coarse information such as the overall shape of face (likely conveyed by low spatial frequencies) might be sufficient enough for detecting a face amid other objects or for identifying one out of a smaller number of alternative faces. In fact, ample evidence ([5, 29, 30, 68]; for a recent review see [7]) has supported the view [69] that task demand (the nature of task) plays a critical role in determining the relative contribution of spectral information in object recognition.

About 3.3 million people in the United States over the age of 40 have impaired vision. By 2020, the aging of society is expected to increase this number by 70% to 5.7 million people [70]. Considering the increase in the low-vision population, it is important to understand how the visual system copes with degraded input. Clinical studies have shown that individuals with low vision find it difficult to carry out daily activities such as reading and face recognition [2, 71]. Indeed, their reading speed is much slower and face recognition is poor compared to the performance of aged-matched, normally sighted individuals [72–78]. Since reading and face recognition are among the most common human visual activities, it is important to understand how the human visual system accomplishes these tasks in the presence of blur. Although our manipulation of field of view and blur might not exactly resemble visual field loss and optical blur experienced by low vision patients, our simulation study may provide insights into the mechanism underlying perceptual process in degraded viewing conditions.

Our results suggested that to compensate for blurry vision, individuals with blurry vision need a larger field of view. Consequently, when these individuals with blurry vision acquire a loss of visual field, its impact would be more detrimental than for individuals with relatively intact acuity. The interaction between blur and field of view is particularly relevant to individuals with low vision whose visual acuity and visual field are both compromised (e.g., AMD, diabetic retinopathy, or glaucoma), highlighting the importance of studying these two properties together. Perhaps, larger spatial and temporal requirements for recognizing blurry objects might be related to slower reading rate or poor recognition performance in patients with low vision. Considering the fact that print sizes commonly used in books and newspapers range from 0.2° to 2° visual angle [79], it is unlikely that reduced field of view (except for the case with severe field loss) limits the recognition of individual letters, thereby slowing down reading speed. However, if identifying individual blurry letters requires longer processing time as demonstrated in the current study, it is highly likely that reading becomes slower under condition of low resolution. It is because a word (even most common three-letter words) is not readable unless its individual letters are identifiable [80]. Furthermore, Kwon and Legge [81] measured the size of the visual span (i.e., the number of letters that can be recognized reliably at a glance) called “uncrowded window”[82] over a wide range of blur levels (from 0.8 c/letter to 2.5 c/letter) including unfiltered letters. They found that the size of visual span, known to play a critical role in reading speed for both normal and low vision [75, 83–85], significantly decreased with increasing blur, suggesting that the size of functional field of view for reading becomes increasingly smaller as blur increases. While these findings collectively suggest that both temporal and spatial requirements of blurry object recognition may play a limiting role in reading or object recognition, this assertion requires more detailed work in the future study.

In summary, we found that field of view and viewing time increased exponentially with increasing blur level. Our findings suggest that while human observers excel at recognizing blurry objects, compensating for blur requires an increase in field of view and viewing time. The need for larger spatial and longer temporal integration for blurry objects may further challenge object recognition in low vision. Our findings are not only important for understanding human object recognition in the presence of blur, but also vital for developing effective rehabilitation or assistive aids for individuals with low vision.

## Supporting Information

S1 File Data file contains all the data for each individual subject for the field of view experiment (letter and face recognition) and the viewing time experiment (letter and face recognition). (XLSX)
