# Correction: Compensation for Blur Requires Increase in Field of View and Viewing Time

**DOI:** 10.1371/journal.pone.0300943

**Published:** 2024-03-15

**Authors:** MiYoung Kwon, Rong Liu, Lillian Chien

In Fig 5B, there is an error with the first parameter value in the best-fit equation. It should be listed as 10903.00e, not 1090.30e. The authors have provided a corrected version of Fig 5 here.

**Fig 5:**
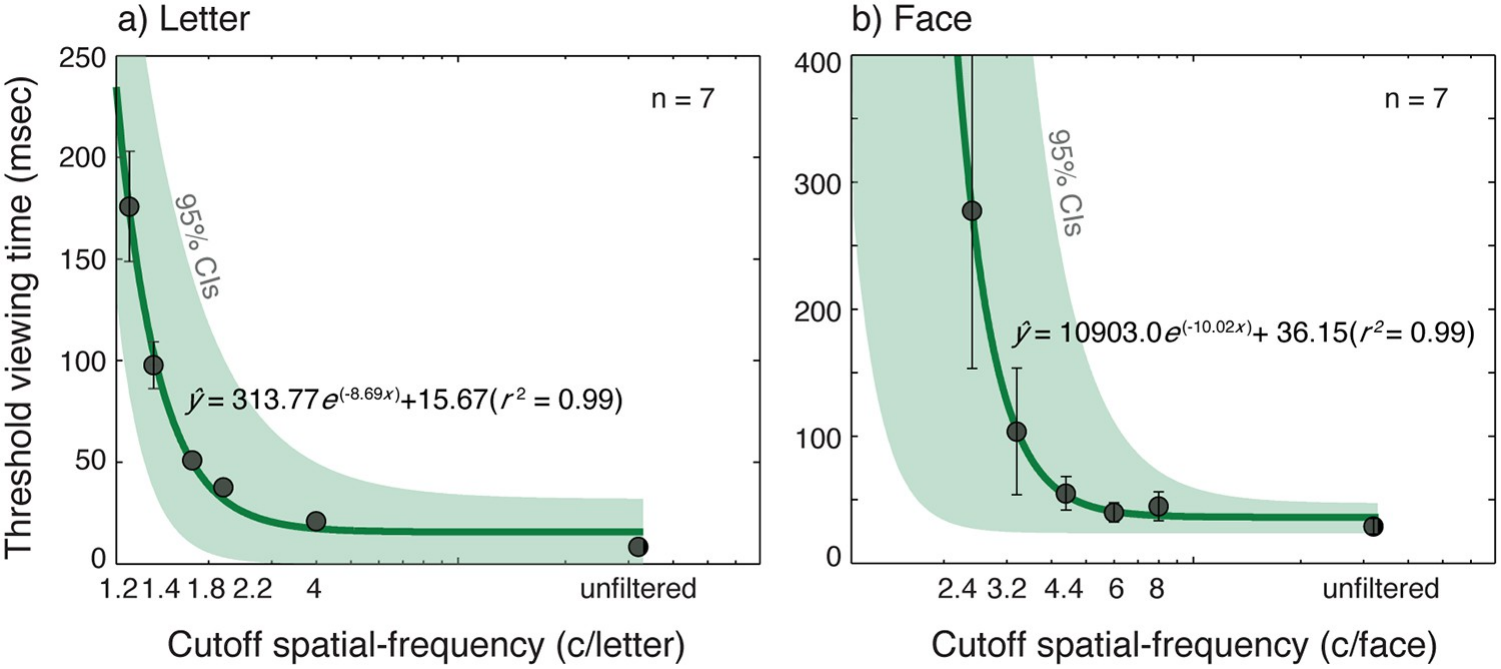
The results from the viewing time study for letter and face. Threshold viewing time (msec) was plotted as a function of cutoff spatial-frequency (blur level). Threshold viewing time was defined as a stimulus exposure time that yielded 80% recognition performance. Stimuli were 26 uppercase letter (a) or 26 celebrity faces (b) with the image size of 2° of visual angle. Each data point (black solid dots) was an average of threshold viewing time across participants (*n* = 7). Data were fitted with the exponential-decay function (Eq 3). The solid lines are the best fits of the model. Error bars represent ±1 SEM. The green shaded areas indicate 95% Confidence Intervals of the fit.
